# Mechanical energy fluctuation in lower limbs during walking in participants with and without total hip replacement

**DOI:** 10.1098/rsos.230041

**Published:** 2023-03-01

**Authors:** St. Fatimah Zahrah Anwar, Ying Wang, Wasim Raza, Graham Arnold, Weijie Wang

**Affiliations:** ^1^ University Department of Orthopaedic and Trauma Surgery, Ninewells Hospital and Medical School, University of Dundee, Dundee DD1 9SY, UK; ^2^ Department of Biomedical Engineering, NED University of Engineering and Technology, LEJ Campus, Karachi, Pakistan

**Keywords:** energy fluctuation, energy recovery, pendulum principle, lower limbs, walking, total hip replacement

## Abstract

Mechanical energy fluctuation of the segments of lower limbs during walking has not been fully investigated. It was hypothesized that the segments may work as a pendulum, i.e. the kinetic and potential energies exchanged out of phase. This study aimed to investigate energy changes and recovery during gait in hip replacement patients. The gait data for 12 participants with total hip replacement and 12 age-matched control was compared. The kinetic, potential and rotative energies for whole lower limb and thigh, calf and foot, were calculated. The effectiveness of a pendulum effect was analysed. Gait parameters (speeds and cadence) were calculated. The results showed that the thigh had significant effectiveness as a pendulum during gait with energy recovery coefficient of approximately 40% while the calf and foot were less like a pendulum during gait. In comparison, energy recoveries of lower limbs in the two groups were not significantly different. If the pelvis was considered as an approximate to the centre of mass, however, the control group had a higher energy recovery than total-hip-replacement group by roughly 10%. This study concluded that, unlike centre of mass energy recovery, the mechanical energy recovery mechanism in the lower limbs during walking is not affected after total hip replacement.

## 1. Introduction

Walking involves moving in a forward direction using the reasonable cost of mechanical and physiological energy expenditure [1,2]. The evolution of the lower limbs and effective utilization of the joint muscles support gait stability. This process conserves potential and kinetic energy and is one of the basics of ‘biological conservation of energy’ [3,4]. Any interruption of the normal gait cycle such as hip osteoarthritis, and the traits of energy preservation of trunk and limb motion will cause higher energy cost [4].

Total hip replacement (THR) is a surgical procedure in which a hip prosthesis replaces the hip joint [5]. The major causal indications for THR are osteoarthritis (OA), which was responsible for 88.4% of hip replacement surgeries in the UK in 2019 [6]. While gait patterns largely improve after THR, they tend not to return to normal, age-matched subjects [7–11]. The biomechanical feature of gait is a valuable clinical tool to differentiate between normal and pathological walking [12], which then affects the treatment and intervention [13–15]. The impact of gait speed on biomechanical variables is essential [16], as pathological individuals tend to walk slower than their healthy counterparts, with cadence and stride length being the determining factors of walking speed [17].

Mechanical energy cost of walking refers to the potential and kinetic energies of the centre of mass (CoM) movement during gait, which can be used to measure gait efficiency [18,19]. Reduced mechanical energy exchange and recovery have been observed in hip OA [20] and abnormalities appear to continue even after THR [21,22]. While higher metabolic energy expenditure has been reported following THR [23–25], abnormalities in mechanical energy exchange and recovery seem to persist following THR [25,26].

**Figure 1.**
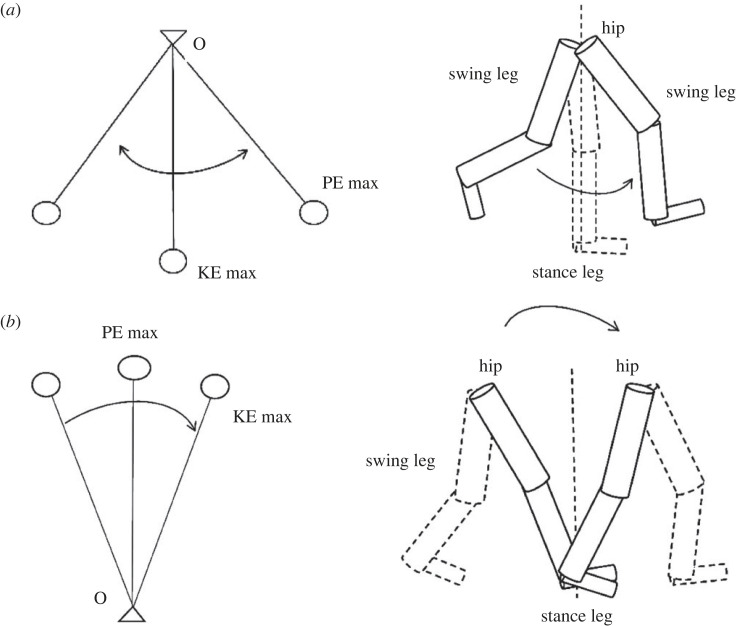
(*a*) The principle of pendulum: kinetic and potential energies exchanged during movement (left). The leg may be similar to a pendulum during swing phase to save total energy. PE and KE: potential energy and kinetic energy (right). (*b*) The principle of inverted pendulum: kinetic and potential energies exchanged during movement (left). The leg may use the principle of inverted pendulum during stance phase (right).

Previous research has attempted to associate the metabolic energy expenditure of walking with walking mechanics. Saunders *et al*. suggested higher vertical displacement would cause higher metabolic cost during gait and the body's effort to minimize acceleration thus smoothing the CoM trajectory [26]. However, Ortega & Farley [27] reported that minimum CoM movement increased metabolic cost [27]. Cavagna *et al*. reported the benefits of mechanical energy exchange of non-flat pendular dynamics, as step-to-step transitions play a major role in dictating the metabolic cost of walking [28,29]. If the mechanical energy exchange is reduced, the muscles will exert more to accelerate and decelerate the CoM during gait [28,30,31], causing higher total internal work following THR [32,33]. This suggests that, to some extent, mechanical energy exchange influences the total energy expenditure during walking. Given the fact that some studies have investigated normal walking using the principle of an inverted pendulum [28,30,34], little research has investigated the limb movements, e.g. the lower limbs moving similarly to a pendulum, i.e. the kinetic and potential energies exchanging out of phase to save energy expenditure when walking. Scientifically, therefore, it is hypothesized that the whole lower limb or some of the segments of the lower limbs could move like a pendulum in swing phase and like an invert pendulum in stance phase during gait (figure 1), which could also be applied to the assessment of gait for THR patients after surgery.

This present study aimed to investigate the changes in the kinetic and potential energies in the segments of lower limbs, and energy recovery during gait for post-THR patients. This study addressed whether the kinetic and potential energies were exchanged efficiently during walking and the differences in terms of energy performance in the lower limbs between the control group and THR patients. Scientifically, research hypotheses were that (i) the lower limbs or some segments may have the potential and kinetic energies exchanged during gait, and (ii) the principle of pendulum may be applied in stance and swing phases. Clinically, the research hypotheses were that (i) THR patients have different gait parameters from the control group, (ii) following THR, the patients would have a lower pendular exchange of mechanical energies in using two mechanical energies than the control group.

## 2. Material and methods

### 2.1. Methodology

This was a prospective and cross-sectional study consisting of gait analysis data from 24 participants with and without THR. Ethical approval was obtained from the local research ethics committee of NHS (09/S1401/65) and the University of Dundee (EB/MC/LET/LN 1384).

### 2.2. Subject selection

All subjects were used irrespective of the side of THR, follow-up period, and age. All subjects were ambulatory. There were 12 post-unilateral THR patients; 5 were in the 1st, 6 were in the 2nd, and 1 participant was in the 6th year after THR surgery when the gait analysis was conducted. Twelve non-THR subjects were selected for the study control group.

### 2.3. Gait analysis

All subjects had undertaken gait analysis using a Vicon® MX motion capture system to collect kinematic data with two Kistler force plates being used to acquire ground reaction forces (GRFs). The data collection was carried out in the Clinical Gait Analysis Lab at the University of Dundee with Vicon Nexus 2.11. The Vicon Plug-in-Gait model was used to process the data to obtain the coordinates for the hip, knee and ankle joints, gait parameters, e.g. walking speeds, cadences, stride length.

Subjects were suitably attired and anthropometric data including weight, height, and segment lengths (e.g. whole limb, upper leg, lower leg and foot segment) were measured. Retro-reflective markers were then attached to the exact anatomical landmarks described in the Vicon manual, including left/right anterior superior iliac (L/RASI), left/right posterior superior iliac (L/RPSI), left/right thigh (L/RTHI), left/right knee (L/RKNE), left/right tibia (L/RTIB), left/right ankle (L/RANK), left/right heel (L/RHEE) and left/right toe (L/RTOE). A Vicon MX camera (Vicon MX40, Oxford, UK) was used to collect marker coordinates at 100 Hz sampling speed. Each participant was asked to walk barefoot at a self-directed speed along a 10 m long walkway. The gait events were manually defined using three points, i.e. foot strike, foot off and next foot strike. The gait parameters were calculated using the marker coordinates and events timing. At least 10 trials were conducted, in which three good trials (e.g. no missing markers or very small gaps in marker trajectories) were selected for analysis. As a result, 33 trials for operated sides and 33 trials for non-operated side from the post-THR group and 35 trials for both legs from the control group were analysed for this study.

### 2.4. Energy calculation

After processing the data in Vicon Nexus, the ASCI (*.csv) files were exported to a custom-built software program using Matlab where various energies were calculated. Energy was calculated frame by frame for a gait cycle, then analysed for the whole gait cycle and for the stance and swing phases, respectively.

The equations used for translational kinetic energy and gravitational potential energy for each participant were:

2.1 $$\mathrm{KE}=\left(\frac{1}{2}\right)mv^{2}$$

and

2.2 PE=mgh.

Where KE is the kinetic energy; PE is the potential energy; *m* is the segment mass; *v* is the velocity of CoM for a segment, e.g. the upper leg, lower leg or foot; *g* is the gravitational constant of approximately 9.81 m/s^2^; and *h* is the height between the CoM of a segment and a reference level on the lab ground. The mass and CoM for each segment were calculated by referring to the relative mass of the whole body and the relative length of the segment [35,36].

As the segment motion includes rotation, rotational kinetic energy (Joule) is also calculated using the equation below:

2.3 $$RKE=\left(\frac{1}{2}\right)I_{c}\omega^{2},$$

where $I_{c}\;$ represents the moment of inertia of the segment about the CoM of a segment, with units kgm^2^; ω is the angular velocity of the segment with units rad/s. *I*_c_ usually is available from references [35,36]. To calculate limb rotations, a vector was placed in each limb/segment from the proximal to distal joints. A related angle between two interval frames in space was defined as the rotative angle of the limb, and then the rotative angular velocity was calculated by dividing the angle with the time duration taken between two interval frames. Therefore, this angle and angular velocity included both flexion/extension and adduction/abduction rotations. The rotation around the long axis of bone was ignored. *I*_c_ usually is estimated as the equation below,

2.4 $$I_{c}=m\rho_{0}^{2}.$$

Where *m* is a segment mass, and *ρ* is the radius of gyration which is equal to the product of a ratio and limb length. The ratios for the thigh, leg and foot are 0.323, 0.302 and 0.475 [36]. For example, a participant has 80 kg in body mass and the thigh mass is estimated as 0.1 × 80 = 8 kg where 0.1 is relative mass for thigh [36]. The thigh length is calculated from hip to knee joint centres, e.g. 0.35 m, and thus *ρ* = 0.35 × 0.323 and *I*_c_ = 8x(0.35 × 0.323)^2^. Using this method, each segment's mass and its moment of inertia was estimated.

In order to assess the efficiency of energy transformation between kinetic and potential energies, a coefficient of energy recovery was used:

2.5 $${\mathrm{Recovery}}_{n}=\frac{\left(\Delta PE+\Delta KE)-\Delta (PE+KE\right)}{\left(\Delta PE+\Delta KE\right)},$$

where ΔKE is the maximum change in kinetic energy, ΔPE is the maximum change in potential energy, and Δ(PE + KE) is the maximum change in the sum of the two energies [34]. Kinetic energy includes translational and rotative energies. The coefficient has a range from 0%−100%, i.e. the worst to best energy exchange rates. Obviously, the higher the coefficient, the better the energies exchanged. As energy is scalar, various forms of energy could be exchanged with each other. Therefore, the translational and rotative forms are permitted to be exchanged. However, the rotative energy is so small compared with the translational one that it was ignored in this study. This equation gives a general form to estimate how potential and kinetic energies exchanged in an object during movement and can be used to estimate energy recovery efficiency for either the pendulum or invert pendulum situations. In fact, a real pendulum is a free movement of pendulum with gravity only and without functioning by muscles and ligament. This study was to investigate to what degree a segment move likes a pendulum, i.e. to what degree a segment uses the principle of pendulum.

In energy calculations, the three segments, i.e. Upper Leg, Lower Leg, and Foot, were calculated separately and together. When the lower limbs were considered as a whole system, the equations were used as below,

2.6 $$X_{\mathrm{CoM}}=\frac{\sum x_{i}m_{i}}{M},$$

2.7 $$Y_{\mathrm{CoM}}=\frac{\sum y_{i}m_{i}}{M}$$

2.8 $$\mathrm{and}\;\;Z_{\mathrm{CoM}}=\frac{\sum z_{i}m_{i}}{M}.$$

Where *X*_CoM_, *Y*_CoM_ and *Z*_CoM_ are the coordinates of whole system, *x_i_*, *y_i_* and *z_i_* the coordinates of each segment CoM, *m_i_* the mass of each segment, and *M* the total mass of the lower limbs.

The calculation was carried out for the factor defined as ‘side’, which resulted in three sides, namely the control, THR operated and THR non-operated sides of the lower limb during gait.

As there were no upper body markers collected, there is no way to calculate the energy for CoM. As an effort, here we calculated the energy in the ‘centre of pelvis’ (CoP) which was defined by the centre of four reflective markers, i.e. L/RASI and L/RPSI in the pelvis [37]. The movement in the CoP could be considered as the approximate to CoM, i.e. assuming that CoP is close to CoM for the whole body, although there are some errors [37].

### 2.5. Statistical analysis

IBM SPSS v. 28 was used to compare differences in energy-related parameters in the control and THR groups. The comparisons were done for gait parameters and energy expenditure from both groups. The statistical methods used were the Independent Sample *T*-Test for comparing demographic data between control and post-THR group and the General Linear Model for comparing the variables among the control, THR-operative and THR-nonoperative sides. In the general linear model, the main effect was the side type, the interactive factor was sex and the covariate factor was BMI (body mass index), i.e. body mass/height^2^. The level of significant difference was set at a *p*-value < 0.05. Bonferroni correction was used as an adjustment for multiple comparisons.

## 3. Results

### 3.1. Demography and gait parameter

The control group comprised 12 subjects, with 25% (3) being male and 75% (9) female. The average age for the control group was 54 years, whereas for the post-THR group it was 56 years (table 1). The post-THR group consisted of 12 subjects, with 6 (50%) males and 6 (50%) females. There was a significant difference in the height and BMI between the two groups as shown in table 1.

**Table 1.** Descriptive statistics between control and patient groups. (*N* = 12 for control and post-THR groups respectively. Sex: control: 9 male 3 female, THR: 6 male and 6 female).

variable	group	mean	std. deviation	range	*p*
age (year)	control	54.42	3.476	48–59	0.386
	post-THR	56.50	7.379	43–65	
body mass (kg)	control	76.433	8.3939	58–86	0.090
	post-THR	85.083	14.6951	54–113	
height (m)	control	1.7236	0.084	1.59–1.87	0.025
	post-THR	1.6492	0.067	1.55–1.77	
BMI	control	25.67	1.40	23–28	0.005
	post-THR	31.40	6.15	20–46	

The results showed that the walking speed, stride length, step length and step width in the THR group were similar to the control group as shown in table 2.

**Table 2.** Comparison of gait parameters between control and patient groups.

information	group	mean	std. error
cadence (steps/minute)	control	112.60	1.41
	op	113.43	1.75
	non-op	113.54	1.75
walking speed (m/s)	control	1.21	0.02
	op	1.17	0.03
	non-op	1.18	0.03
stance % of gait cycle	control	60.59	0.33
	op	60.56	0.41
	non-op	61.26	0.41
stride length (m)	control	1.30	0.02
	op	1.24	0.02
	non-op	1.24	0.02
step length (m)	control	0.65	0.01
	op	0.62	0.01
	non-op	0.62	0.01
step width (m)	control	0.18	0.00
	op	0.16	0.01
	non-op	0.16	0.01

^a^Covariates appearing in the model are evaluated at the following values: BMI = 28.4662.

^b^Sex was input as an interactive factor.

^c^Adjustment for multiple comparisons: Bonferroni.

^d^All *p* > 0.05.

### 3.2. Segment energy

Altogether, the lower limb of the control group participants had similar kinetic energy to post-THR participants (table 3). When dividing them into three segments, the energy recovery in the control group was statistically lower compared to post-THR participants in the lower leg segment (table 4). There was no significant difference in kinetic and potential energy and energy recovery in both the upper leg and foot segments (tables 5 and 6). As the rotative energy ranges were mean 0.0097 and s.d. 0.0081 joules and translational energy ranges were mean 11.785 and s.d. 3.547 joules, therefore the rotative ones were ignored in this study.

**Table 3.** Comparison of energy on whole lower limb segment during whole cycle.

type of energy	group	mean	std. error
rangeKE (joule)	control	26.64	1.13
	operated	25.59	1.40
	non-operated	26.24	1.40
rangePE (joule)	control	5.66	0.18
	operated	5.84	0.22
	non-operated	6.27	0.22
recoveryCof %	control	9.47	0.38
	operated	10.62	0.48
	non-operated	9.87	0.48

^a^Covariates appearing in the model are evaluated at the following values: BMI = 28.4662.

^b^Sex was input as an interactive factor.

^c^Adjustment for multiple comparisons: Bonferroni.

^d^All *p* > 0.05.

^e^**KE**, kinetic energy; **PE**, potential energy; **RE**, rotational energy; **RecoveryCof**, energy recovery coefficient.

**Table 4.** Comparison of energy on upper leg segment during whole gait cycle.

segment	type of energy	group	mean	std. error
upper leg	rangeKE (joule)	control	11.11	0.47
		operated	10.67	0.59
		non-operated	11.04	0.59
	rangePE (joule)	control	3.64	0.14
		operated	4.09	0.17
		non-operated	4.05	0.17
	recoveryCof (%)	control	39.12	0.84
		operated	42.22	1.05
		non-operated	40.08	1.05

^a^Covariates appearing in the model are evaluated at the following values: BMI = 28.4662.

^b^Sex was input as an interactive factor.

^c^Adjustment for multiple comparisons: Bonferroni.

^d^KE, kinetic energy; PE, potential energy; RecoveryCof, energy recovery coefficient.

^3^all *p* > 0.05.

**Table 5.** Comparison of energy on lower leg segment during whole gait cycle.

segment	type of energy	group	mean	std. error	pairwise comparison	
					pairing	*p*-value^b^
lower leg	rangeKE (joule)	control	11.20	0.47	control versus op	1
		operated	10.87	0.58	control versus non-op	1.000
		non-operated	11.41	0.58	op versus non-op	1
	rangePE (joule)	control	2.16	0.06	control versus op	0.423
		operated	2.17	0.07	control versus non-op	0.084
		non-operated	2.32	0.07	op versus non-op	0.901
	recoveryCof (%)	control	1.36*	0.18	control versus op	<0.001
		operated	3.01*	0.23	control versus non-op	<0.005
		non-operated	2.37*	0.23	op versus non-op	0.118

^a^Covariates appearing in the model are evaluated at the following values: BMI = 28.4662.

^b^Sex was input as an interactive factor.

^c^Adjustment for multiple comparisons: Bonferroni.

^d^**KE**, kinetic energy; **PE**, potential energy; **RecoveryCof**, energy recovery coefficient.

**Table 6.** Comparison of energy on foot segment during whole gait cycle.

segment	type of energy	group	mean	std. error
foot	rangeKE (joule)	control	12.04	0.46
		operated	11.21	0.57
		non-operated	11.70	0.57
	rangePE (joule)	control	1.74	0.03
		operated	1.71	0.04
		non-operated	1.71	0.04
	recoveryCof (%)	control	8.73	0.21
		operated	8.60	0.26
		non-operated	8.38	0.26

^a^Covariates appearing in the model are evaluated at the following values: BMI = 28.4662.

^b^Sex was input as an interactive factor.

^c^Adjustment for multiple comparisons: Bonferroni.

^d^**KE**, kinetic energy; **PE**, potential energy; **RecoveryCof**, energy recovery coefficient.

^e^all pairs *p* > 0.05.

**Figure 2.**
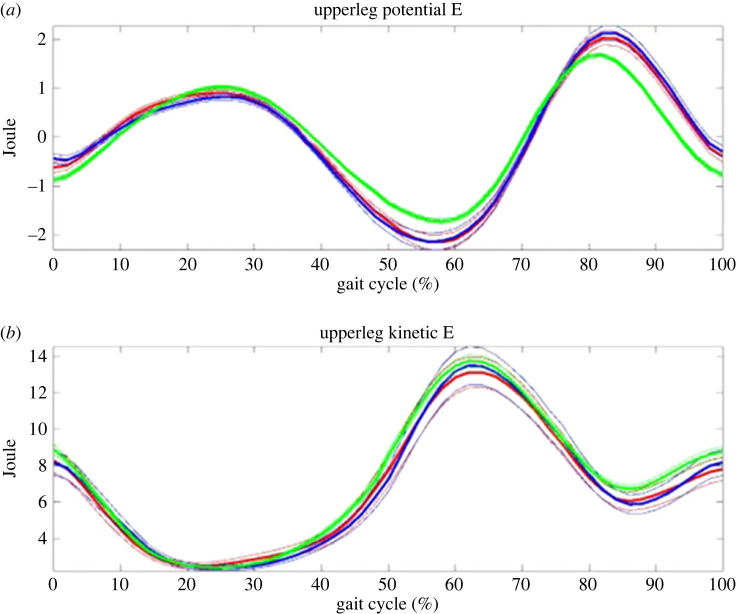
The upper leg: potential (upper) and kinetic (lower) energies change during gait. Kinetic and potential energies exchanged out of phase, and thus the principle of pendulum was applied. Note: green line—control; blue line—THR non-operative side; red line—THR operative side; thick lines are mean and thin ones are standard error of mean; the number of trials was 72 for control including both sides, and 33 for operative side and non-operative sides, respectively; please note that the potential energies have been normalized by a reference height as self-mean; the roughly first 60% of gait cycle is the stance phase and the remaining 40% is the swing phase; all notes are the same in the following figures.

**Figure 3.**
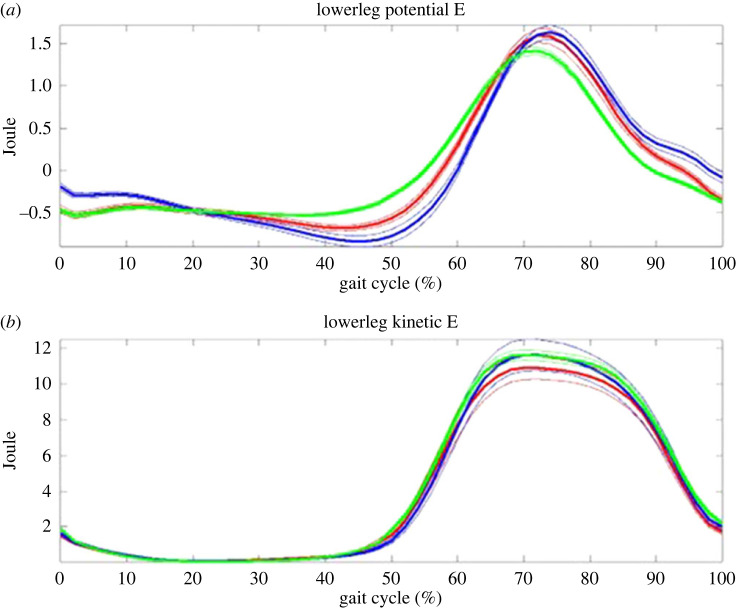
The lower leg: the kinetic (lower) and potential (upper) energies change during gait. Kinetic and potential energies exchanged in phase, and thus the principle of pendulum was not applied. Note: green line—control; blue line—THR non-operative side; red line—THR operative side.

**Figure 4.**
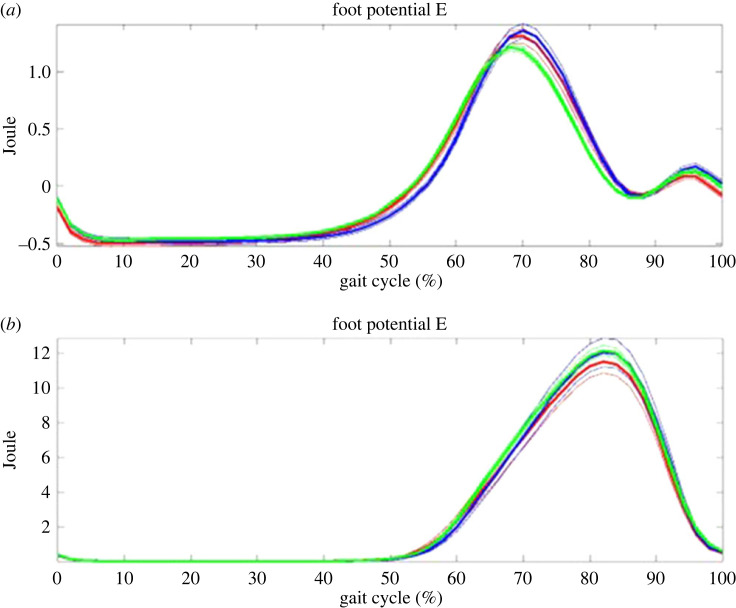
The foot: the kinetic (upper) and potential (lower) energies change during gait. Kinetic and potential energies exchanged nearly in phase, and thus the principle of pendulum was not applied in the foot.

**Figure 5.**
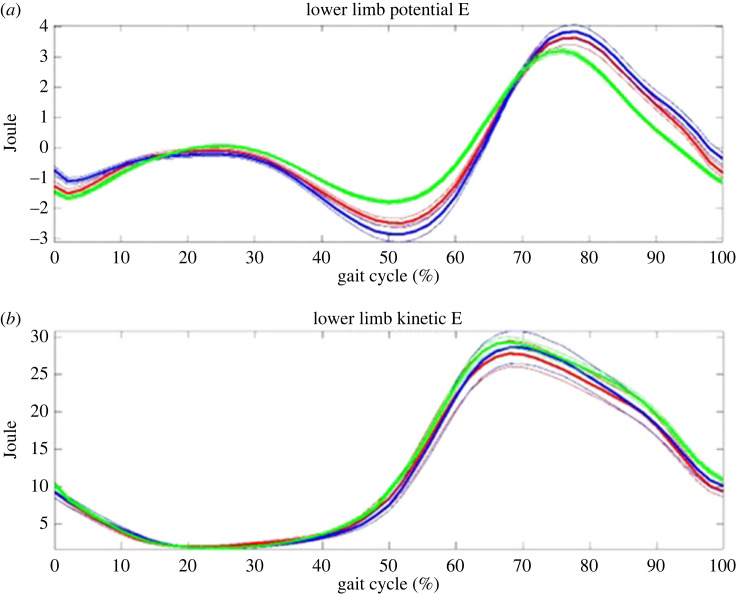
The lower limb: the kinetic (lower) and potential (upper) energies change during gait. Kinetic and potential energies exchanged nearly in phase, and thus the principle of pendulum was not applied in the lower limb. Note: green line—control; blue line—THR non-operative side; red line—THR operative side.

According to the equations (1–4), each trial from both groups was produced. Figures 2–5 show all trial curves for three segments in the lower limbs, respectively. From the figures, it is obvious that the upper leg has the kinetic and potential energies exchanging very well, i.e. KE increased/decreased while PE decreased/increased out of phase, But the lower leg and foot were in phase, i.e. not as efficient as the upper leg in energy recovery.

### 3.3. Energy in the stance and swing phases

The stance and swing phases were analysed respectively. The results showed that the upper leg has greater energy recovery than the lower leg and foot in both stance and swing phases in tables 7 and 8.

**Table 7.** Comparison of the energy in swing phase for different segments.

segment	dependent variable	type	mean	std. error	95% confidence interval	
					lower bound	upper bound
upper leg	swing range PE (joule)	control	3.56	0.15	3.25	3.86
		THR-op	3.90	0.19	3.53	4.28
		THR-non-op	3.81	0.19	3.43	4.18
	swing range KE (joule)	control	7.15	0.28	6.60	7.69
		THR-op	7.29	0.34	6.61	7.97
		THR-non-op	7.66	0.34	6.98	8.34
	swing recoveryCof%	control	54.04	1.50	51.07	57.01
		THR-op	56.80	1.86	53.11	60.48
		THR-non-op	55.79	1.86	52.11	59.47
lower leg	swing range PE (Joule)	control	1.88	0.06	1.75	2.01
		THR-op	1.80	0.08	1.65	1.96
		THR-non-op	1.72	0.08	1.56	1.87
	swing range KE (joule)	control	9.12	0.39	8.35	9.89
		THR-op	9.29	0.48	8.33	10.24
		THR-non-op	9.53	0.48	8.58	10.49
	swing recoveryCof%	control	1.09	0.29	0.52	1.65
		THR-op	1.39	0.36	0.69	2.09
		THR-non-op	2.02	0.36	1.31	2.72
foot	swing range PE (Joule)	control	1.43	0.03	1.37	1.49
		THR-op	1.41	0.04	1.33	1.48
		THR-non-op	1.39	0.04	1.31	1.46
	swing range KE (joule)	control	11.49	0.44	10.61	12.36
		THR-op	10.77	0.55	9.68	11.85
		THR-non-op	11.19	0.55	10.10	12.27
	swing recoveryCof%	control	10.47	0.28	9.92	11.01
		THR-op	9.67	0.34	8.99	10.34
		THR-non-op	10.00	0.34	9.32	10.68

^a^Covariates appearing in the model are evaluated at the following values: BM = 28.4662.

^b^Sex was input as an interactive factor.

^c^Adjustment for multiple comparisons: Bonferroni.

^d^All pairs have *p* > 0.05.

**Table 8.** Comparison of the energy in stance phase for different segments.

segment	dependent variable	type	mean	std. error
upper leg	stance range PE (joule)	control	2.80	0.11
		THR-op	3.01	0.13
		THR-non-op	2.91	0.13
	stance range KE (joule)	control	10.10	0.48
		THR-op	10.05	0.59
		THR-non-op	10.60	0.59
	stance recoveryCof%	control	41.45	1.00
		THR-op	41.71	1.23
		THR-non-op	37.99	1.23
lower leg	stance range PE (joule)	control	0.93	0.04
		THR-op	0.89	0.05
		THR-non-op	1.02	0.05
	stance range KE (joule)	control	6.65	0.34
		THR-op	6.52	0.42
		THR-non-op	7.01	0.42
	stance recoveryCof%	control	1.83	0.38
		THR-op	2.35	0.47
		THR-non-op	3.31	0.47
foot	stance range PE (joule)	control	0.86	0.03
		THR-op	0.88	0.03
		THR-non-op	0.84	0.03
	stance range KE (joule)	control	1.55	0.11
		THR-op	1.62	0.13
		THR-non-op	1.65	0.13
	stance recoveryCof%	control	0.04	0.02
		THR-op	0.13	0.02
		THR-non-op	0.05	0.02

^a^Covariates appearing in the model are evaluated at the following values: BMI = 28.4662.

^b^Sex was input as an interactive factor.

^c^Adjustment for multiple comparisons: Bonferroni.

^a^All pairs have *p* > 0.05.

### 3.4. Energy changes in CoP

**Figure 6.**
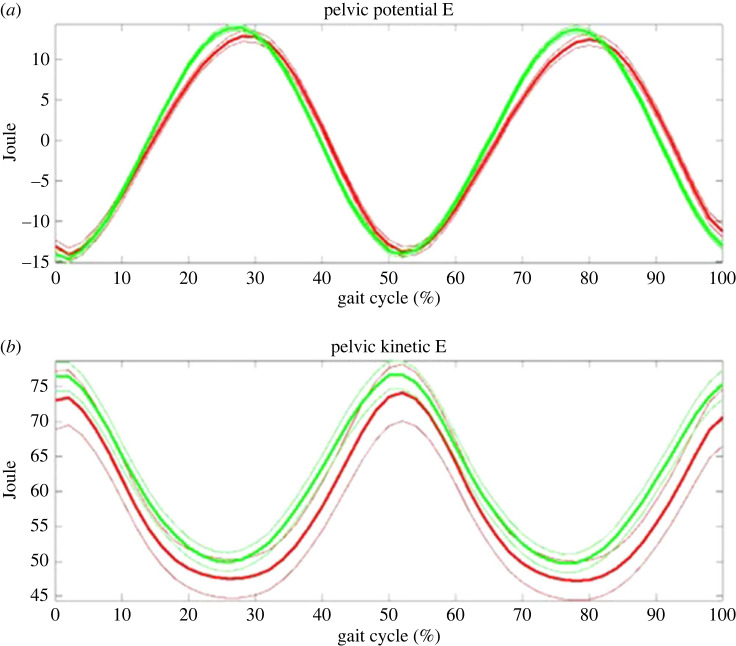
The centre of pelvis as an approximate to CoM: the kinetic (lower) and potential (upper) energy changes during gait. Kinetic and potential energies exchanged at the perfect phase, and thus the principle of pendulum was applied in whole body. Note: green line—control; red line—THR group. The thick line is mean and the thin line standard error of mean.

The results in CoP showed nearly perfect energy recovery between the potential and kinetic forms exchanged during gait in table 9 and figure 6. Moreover, the control group showed better energy recovery than the THR group by a roughly 10% higher recovery coefficient.

**Table 9.** Comparison of energy recoveries in the CoP for the THR and control groups.

dependent variable	group	mean	std. error	95% confidence interval		
				lower bound	upper bound	*p*
range KE (joule)	control	29.31	1.51	26.33	32.29	0.278
	THR	31.73	1.38	28.99	34.46	
range PE (joule)	control	31.80	0.97	29.88	33.71	0.241
	THR	30.12	0.89	28.36	31.88	
recoveryCof (%)	control	79.84	1.07	77.72	81.97	<0.001*
	THR	70.79	0.99	68.84	72.74	

^a^Covariates appearing in the model are evaluated at the following values: BMI = 28.4772.

^b^Adjustment for multiple comparisons: Bonferroni.

^c^BMI was input as covariate and sex as an interactive factor.

## 4. Discussion

### 4.1. Gait parameters

Post-THR patients exhibited similar gait parameters to control subjects in this study. The mean walking speed of 1.12 m s^−1^ of post-THR patients, however, is much faster compared to those reported in previous studies [8,22,38]. Interestingly, the walking speed of post-THR patients in the present study is similar to those of healthy adults in the same age range in a study by Monaco *et al*. [39]. Although age could be the factor in reduced walking speed [7,22], even when choosing the younger post-THR patients (mean age: 56 years) and appropriately matching them with a similar age control group, the control group still showed similar walking speed (table 2). While Miki *et al*. [40] described normalization of walking speed after 1 year of unilateral THR, the present study with a follow-up range of 1–6 years and other studies have also reported similar results of reduced walking velocity after longer follow-up [11,41,42].

The changes in stride length in the post-THR group have not been observed in this study, although previous studies have mentioned that the reduction of stride length may be caused by a reduced hip RoM [8–10,33,41], in which Beaulieu *et al*. also described reduced peak hip extension [7]. As a result, individuals may need to lift the lower limb higher, thus causing higher energy expenditure because of higher vertical displacement [26,43]. The present study, however, did not find difference in stride lengths, nor any increase in mechanical energy expenditure. Pain [44], muscle weakness [9,41], and soft tissue damage [41] could also cause a reduction in the hip RoM and the consistent shorter stride length reported.

Previously, narrower step width has been reported to be linked to higher work because of the circumduction of the swing leg around the stance leg, which increases metabolic cost [45]. In contrast, Metcalfe *et al*. [38], reported wider step width compared to controls. However, there is no significant difference between both groups in our study (table 2), its implication in a clinical setting may not be significant.

While the present study observed a significant difference in height between the two groups, this did not seem to affect the mechanical energy exchange in both groups (tables 3–6) after BMI was used as covariates and sex as interactive factor in statistical analysis.

The results when comparing the operated and non-operated limb's gait parameters in this investigation showed no significant differences, suggesting good biomechanical recovery following THR. Previous work by Connor *et al*. [46] found similar hip contact force profiles between operated and non-operated limbs, which also suggests a symmetrical biomechanical recovery in unilateral THR. Bennett *et al*. [8] also reported symmetrical kinematic variables in a 10-year follow up, which supports the premise that kinematic symmetry could be achieved in the long term following THR.

In contrast, Foucher and Wimmer found an increased abduction moment in non-operated hips even up to a year after THR, which may lead to abnormalities in gait spatiotemporal parameters [47]. Our findings of equal gait parameters between operated and non-operated limbs may be due to a longer period of recovery, which may also be the case for previous studies [8,46].

### 4.2. Mechanical energy

Mechanical energy expenditure mirrors an individual's functional performance capability [2,36]. Queen *et al.* [20] used a whole-body model to analyse OA patients' walking, using the integral of ground reaction force to estimate energy fluctuation in CoM. They reported reduced energy recovery in any joint with OA, especially with hip and ankle OA, where the energy recovery was never achieved as in asymptomatic subjects. However, they did not consider any segments in terms of energy recovery. Hip OA has a high chance of disturbing energy exchange mainly because of the reduced RoM in hip extension during terminal stance [48]. This may restrict the rise and fall of the CoM, which then reduces the amount of potential energy stored and disrupts the energy curves as it changes the timing of pushoff relative to the timing of the peak potential energy [20]. In theory, any disruption of the normal gait cycle and energy-conserving traits of body motion would cause an increase in energy expenditure [4,26], thus it is crucial to revert the energy recovery values closer to asymptomatic individuals to decrease muscular effort, pain, and tiredness during gait.

Following THR, Loizeau *et al*. showed there was still a reduction in mechanical energies at the hip and knees, even in non-operated limbs [22]. Although energy expenditure was reduced when compared to pre-operative data, it was still not within normal limits [23,32]. The present study did not support this, as the results show similar values in potential and energy recovery between post-THR and control subjects (tables 3–6), even between the operated and non-operated legs (tables 3–6). This was the opposite of the study hypothesis, where the mechanical energy expenditure would be higher, thus reducing mechanical energy exchange and recovery will be observed following THR. This suggests that following THR, energy exchange and recovery can obtain similar values as those in age-matched, asymptomatic subjects.

During gait, walking speed was one of the clinical indicators and a significant measure of functional capacity in the elderly, with reduced speed associated with a higher risk of poor health-related outcomes [1,49]. A study by Wang *et al*. [34 found that a comfortable gait speed has the optimum mechanical energy exchange and recovery. While it is difficult to directly associate physiological energy cost with mechanical energy exchange and recovery, it was found that a comfortable walking speed has higher (better) energy exchange and recovery [34]. A previous study by Huang and Foucher reported low mechanical energy exchange, with a positive relationship between fatigue and mechanical energy exchange in THR patients [21]. The association between the two may have been caused by patients doing compensatory gait to reduce fatigue [4], where they reduce the body's motion during gait [21]. This was not correlated, as our results showed similar walking speed and kinetic energy exchange in post-THR patients (tables 2–6).

In terms of energy exchanges in the lower limb segments, this study has found that the upper leg has higher energy recovery, roughly 40%, compared to the lower leg and foot, roughly less than 10%. In other words, the upper leg works better as the principle of a pendulum than the lower leg and foot. To our best knowledge, this is the first study to report this finding. Future research on the lower limb segments is recommended to further investigate the energy recovery mechanism in each segment, thus providing better rehabilitation following THR.

It was also found that the ‘centre of pelvis’ (CoP), as an approximate to CoM, has an energy recovery coefficient as high as roughly 70%, and also that the control group was better than the THR by roughly 10% (table 9). This result shows that as whole body, the control group has a greater capacity than the THR in using energy, although the lower limbs did not show significantly difference in energy recovery. This finding also brings in another hypothesis that the upper limbs, trunk, neck and head may contribute to the energy exchange mechanism by using their coordinated movements to the walking.

### 4.3. Limitations

There were no pre-operative gait data for the THR group, and thus it is impossible to compare pre- and post-operative gait patterns. In the calculation of mechanical energy, the segment mass for post-THR patients was unclear, thus normal anthropometric proportions were used. In addition, THR data covers a long period from 1–6 years, and it is not clear if this long period would bring in any bias. When estimating the CoM movements, we fully understand that the centre of pelvis used is not the CoM for whole body, although this approximate gave us indication on the CoM. These shortcomings also indicate the directions for future study.

## 5. Conclusion

The present study was conducted to analyse the energy exchange mechanism and recovery in the lower limbs of post-THR patients during walking. The THR and control groups had similar walking speed, stride length, wider step width, and both groups had similar energy recovery in limb segments. This suggested that the mechanical energy recovery mechanism in the lower limbs during walking was comparable to those in age-matched, asymptomatic individuals. In terms of principle of a pendulum, the upper leg has a significant effect on gait while the lower leg, foot and whole lower limb are not significant. Also, the upper leg has better energy recovery in swing phase than in stance phase. When the centre of pelvis was analysed for the whole body, the control group is better at energy recovery than the THR by roughly 10%, indicating that the THR gait still has room to be improved with respect to whole-body movement.

Regarding the comparison of energy recovery in the operated side and non-operated side of the lower limbs of THR patients, there was no significant difference in all segments, suggesting good biomechanical recovery.

## Data Availability

The data are available from the Dryad Digital Repository: https://doi.org/10.5061/dryad.r4xgxd2fr [50].
